# Publishing activities improves undergraduate biology education

**DOI:** 10.1093/femsle/fny099

**Published:** 2018-04-17

**Authors:** Michelle K Smith

**Affiliations:** School of Biology and Ecology, University of Maine, 5751 Murray Hall, Orono, ME 04469-5751, USA

**Keywords:** education, active-learning, undergraduate, teaching collaboration

## Abstract

To improve undergraduate biology education, there is an urgent need for biology instructors to publish their innovative active-learning instructional materials in peer-reviewed journals. To do this, instructors can measure student knowledge about a variety of biology concepts, iteratively design activities, explore student learning outcomes and publish the results. Creating a set of well-vetted activities, searchable through a journal interface, saves other instructors time and encourages the use of active-learning instructional practices. For authors, these publications offer new opportunities to collaborate and can provide evidence of a commitment to using active-learning instructional techniques in the classroom.

## INTRODUCTION

Using active-learning instructional techniques increases student learning and decreases the failure rate in STEM classes (Freeman *et al.*^2014^). As a result, several reports have called for a revolution in how undergraduate biology is taught, moving away from a lecture-only approach to one in which students are engaging in problem-solving activities, having peer discussions and asking authentic questions in the classroom (AAAS 2011; President's Council of Advisors on Science and Technology 2012; European Commission High Level Group 2013; National Research Council 2013). Many biology instructors are answering these calls by developing new classroom activities and evaluating their quality through assessments of student learning.

To develop new classroom activities, biology instructors often collect evidence about student conceptual difficulties using a variety of response validated assessment instruments (Smith, Wood and Knight 2008; Nehm *et al.*^2012^; Paustian *et al.*^2017^), develop instructional materials and assessment questions that address the conceptual difficulties, collect evidence about student learning and make iterative revisions. For example, a group of instructors from multiple institutions asked their students about the impact of a premature stop codon on DNA replication, transcription and translation (Prevost, Smith and Knight 2016); learned that many students had a mixed understanding of the central dogma of biology; developed an active-learning case study activity that addressed the identified conceptual difficulties; and measured improvement in student learning using multiple assessment questions (Pelletreau *et al.*^2016^).

Arguably, developing classroom activities results in important intellectual contributions that are an indication of a commitment to using evidence-based active-learning teaching techniques. This commentary encourages biology instructors to publish their activities in peer-reviewed journals such as *FEMS Microbiology Letters* (https://academic.oup.com/femsle/issue/363/16), *CourseSource* (https://www.coursesource.org/) and the *Journal of Microbiology and Biology Education* (http://www.asmscience.org/content/journal/jmbe). These publications can be used to help other instructors overcome barriers to using active-learning, afford an opportunity to collaborate with colleagues on issues of teaching and learning and provide documented evidence for scholarly activities around teaching.

## PUBLISHING ACTIVITIES PROMOTES ACTIVE-LEARNING

Classroom observation data and faculty surveys indicate that, while on the decline, lecture is a predominant instructional behavior in undergraduate classrooms (Eagan 2016; Manduca *et al.*^2017^; Stains *et al.*^2018^). When undergraduate instructors are asked about barriers to incorporating active-learning into their classrooms, they often talk about a lack of time, incentives and professional development opportunities (Silverthorn, Thorn and Svinicki 2006; Henderson and Dancy 2007; Brownell and Tanner 2012). Having access to high-quality activities would minimize some of these barriers and could help more instructors get to a place where teaching with well-vetted active-learning activities is the norm in undergraduate biology classrooms.

One option currently available to instructors is to search biology concepts of interest on the internet, such as microorganism replication, and explore the activities that exist. However, sorting through the activities takes time and because it is rare to get peer review on instructional materials, it can be difficult to determine which activities will be successful. Furthermore, there are often errors, directions that are difficult to follow and links that have not been updated. Instead, if instructors could search journals with peer-reviewed biology activities that have been taught in undergraduate classrooms and are searchable by key concepts, chances are higher that they would find activities with fewer errors, more evidence of student learning and materials that are ready to use or easy to adapt. Currently, many journals are eager to expand the activities they publish and view their role as coaches in the peer-review and publication process (Blum *et al.*^2018^).

## DEVELOPING PUBLISHABLE ACTIVITIES ENCOURAGES NEW COLLABORATIONS

Scientific research collaborations are ubiquitous, critical to discovery and a celebrated part of the field (National Research Council 2015). Moreover, these benefits can extend to teaching collaborations where colleagues develop activities and publish their findings. Together groups of instructors can collect evidence about student conceptual difficulties, share experiences and ideas, write activities and assessment questions, collect evidence about student learning, make iterative revisions and publish their findings (Pelletreau *et al.*^2016^; Smith *et al.*^2018^). Furthermore, recent work has shown that when faculty collaborate on the development of an activity, teach it in their classrooms, and co-author a publication, they are more likely to use active-learning instructional techniques (Pelletreau *et al.*, 2018).

There are a variety of ways to find collaborators. Instructors can often collaborate with colleagues at their institution who teach the same course at a different time or teach courses that come before or after their courses. Teaching collaborators also frequently meet at professional development events. For example, participants in the Summer Institutes on Scientific Teaching learn about active-learning, assessment and inclusive teaching in a week-long immersive professional development institute (Pfund *et al.*^2009^). The participants also develop a ‘Teachable Tidbit’—an instructional unit to be used at their home institution (Wood and Handelsman 2004). Several groups of instructors have taught their Teachable Tidbits, made iterative changes based on student learning data and published manuscripts (Hoskinson *et al.*^2014^; Sestero *et al.*^2014^; Emtage *et al.*^2016^; Freeman *et al.*^2017^). Professional society meetings are also great ways to start teaching collaborations. Often there are roundtable or ‘dine and discuss’ events where developing activities can be the focus of the conversation.

Publishing activities also offer an opportunity to collaborate with graduate students and postdocs. The chance to design and publish activities can provide pedagogical training that is often highly sought after by graduate students and postdocs (Bok 2013), and help prepare them for a variety of careers that often include teaching. Publications about instructional activities can enhance curricula vitae, provide evidence of excellence in teaching, help prepare for the teaching demonstration portion of academic interviews (Smith, Wenderoth and Tyler 2013) and be used as examples in teaching philosophy statements that are often requested in job applications.

## PUBLISHING ACTIVITIES IS EVIDENCE OF A COMMITMENT TO HIGH-QUALITY TEACHING

Recently, there have been several movements to rethink the way teaching effectiveness is evaluated for faculty at the undergraduate level (Sursock 2015; Dennin *et al.*^2017^). Notably, there are generally agreed upon metrics for evaluating research success such as the number and prestige of journal articles and the value of awarded grants. But evaluating teaching expertise can be more difficult because there are fewer quantitative metrics and ones that are commonly used, such as student teaching evaluations, are subject to bias and not necessarily tied to student learning (Centra and Gaubatz 2000; Clayson 2009; Braga, Paccagnella and Pellizzari 2014; Boring, Ottoboni and Stark 2016).

However, if biology faculty publish their activities in peer-reviewed journals, these publications could be used as evidence for effective teaching. For example, instructors can list these publications in the Peer-Reviewed Articles section of their curricula vitae and then report more information about the instructional materials, student learning data and iterative revision process in the teaching section of their tenure and promotion paperwork. Instructors can also consider having someone observe their class when they teach activities they plan to publish using tools such as the Teaching Dimensions Observation Protocol (Hora, Oleson and Ferrare 2013), Classroom Observation Protocol for Undergraduate STEM (Smith *et al.*^2013^) or Practical Observation Rubric To Assess Active Learning (Eddy, Converse and Wenderoth 2015). These observations can provide important data about instructional practices that can be used in the journal publication and as documentation of the use of innovative instructional practices.

Publications about activities could also send a powerful message to prospective students and their families about the value an institution puts on teaching and the types of learning experiences that will occur in the classroom. Departments can highlight teaching accomplishments using similar metrics to research achievements to show how faculty are using student learning data to improve their own teaching and sharing their innovations with a broader audience.

## CONCLUSION

Think about your most effective active-learning activities and consider writing them up for publication. Writing the article will help you reflect on the activity, hold you accountable to evaluating its impact on students and likely improve the quality of the activity for the next time you teach it. Because the writing style is often more similar to a methods paper than a research article, make sure to write about your activities in a way that can be easily replicated by a broad audience. Publishing activities provide benefits to other instructors and the experience may provide an opportunity to form new collaborations centered on teaching. You will also send a powerful message that effective teaching in biology is an important intellectual endeavor that is worthy of being shared and highlighted by your institution.

## References

[bib1] American Association for the Advancement of Science Vision and Change in Undergraduate Biology Education: A Call to Action. Washington, DC: national science foundation, 2011.

[bib2] Blum JE, Knight K, Smith MK Why publish your class activities in CourseSource? 2018 http://genestogenomes.org/why-publish-your-class-activities-in-coursesource/ (30 January 2018, date last accessed).

[bib3] Bok D We Must Prepare Ph.D. Students for the Complicated Art of Teaching. Chronicle of Higher Education, 2013 https://www.chronicle.com/article/We-Must-Prepare-PhD-Students/142893 (30 January 2018, date last accessed).

[bib4] Boring A, Ottoboni K, Stark PB Student evaluations of teaching (mostly) do not measure teaching effectiveness. ScienceOpen Res 2016;1–11.

[bib5] Braga M, Paccagnella M, Pellizzari M Evaluating students’ evaluations of professors. Econ Educ Rev 2014;41:71–88.

[bib6] Brownell SE, Tanner KD Barriers to faculty pedagogical change: lack of training, time, incentives, and…tensions with professional identity? Cell Biol Educ 2012;11:339–46. 10.1187/cbe.12-09-0163 PMC3516788 23222828

[bib7] Centra JA, Gaubatz NB Is there gender bias in student evaluations of teaching? J High Educ 2000;71:17–33.

[bib8] Clayson DE Student evaluations of teaching: are they related to what students learn? A meta-analysis and review of the literature. J Mark Educ 2009;31:16–30.

[bib9] Dennin M, Schultz ZD, Feig A Aligning practice to policies: changing the culture to recognize and reward teaching at research universities. CBE-Life Sci Educ 2017;16:4 es5. 10.1187/cbe.17-02-0032 PMC5749974 29196430

[bib10] Eagan K Becoming More Student-Centered? An Examination of Faculty Teaching Practices across STEM and non-STEM Disciplines between 2004 and 2014. (Alfred P. Sloan Foundation, Los Angeles: Higher Education Research Institute 2016).

[bib11] Eddy SL, Converse M, Wenderoth MP PORTAAL: a classroom observation tool assessing evidence-based teaching practices for active learning in large science, technology, engineering, and mathematics classes. Cell Biol Educ 2015;14:ar23. 10.1187/cbe.14-06-0095 PMC4477739 26033871

[bib12] Emtage L, Bradbury L, Coleman N Cell signaling pathways - a case study approach. CourseSource 2016;3:1–7.28936469

[bib13] European Commission High Level Group on the Modernization of Higher Education Report to the European Commission on Improving the Quality of Teaching and Learning in Europe's Higher Education Institutions Brussels. 2013.

[bib14] Freeman PL, Maki JA, Thoemke KR Evaluating the Quick Fix: Weight loss drugs and cellular respiration. CourseSource 2017 https://doi.org/10.24918/cs.2017.17(26 April 2018, date last accessed).

[bib15] Freeman S, Eddy SL, McDonough M Active learning increases student performance in science, engineering, and mathematics. P Natl Acad Sci USA 2014;111:8410–5. 10.1073/pnas.1319030111 PMC4060654 24821756

[bib16] Henderson C, Dancy MH Barriers to the use of research-based instructional strategies: the influence of both individual and situational characteristics. Phys Rev ST Phys Educ Res 2007;3:010103.

[bib17] Hora MT, Oleson A, Ferrare JJ Teaching Dimensions Observation Protocol (TDOP) User's Manual. University of Wisconsin-Madison 2013. http://tdop.wceruw.org/Document/TDOP-Users-Guide.pdf (30 January 2018, date last accessed).

[bib18] Hoskinson AM, Conner L, Leigh MB Coevolution or not? Crossbills, squirrels and pinecones. CourseSource 2014;1:1–8.

[bib19] Manduca CA, Iverson ER, Luxenberg M Improving undergraduate STEM education: the efficacy of discipline-based professional development. Sci Adv 2017;3:e1600193. 28246629 10.1126/sciadv.1600193 PMC5310824

[bib20] National Research Council Enhancing the Effectiveness of Team Science. Washington, DC: The National Academies Press, 2015 https://doi.org/10.17226/19007. 26247083

[bib21] National Research Council BIO2010: Transforming Undergraduate Education for Future Research Biologists. Washington, DC: National Research Council, 2013.

[bib22] Nehm RH, Beggrow EP, Opfer JE Reasoning about natural selection: diagnosing contextual competency using the ACORNS instrument. Am Biol Teacher 2012;74:92–8.

[bib23] Paustian TD, Briggs AG, Brennan RE Development, validation, and application of the microbiology concept inventory. J Microbiol Biol Educ 2017;18:3. 10.1128/jmbe.v18i3.1320 PMC5976036 29854042

[bib24] Pelletreau KN, Andrews T, Armstrong N A clicker-based case study that untangles student thinking about the processes in the central dogma. CourseSource 2016 https://doi.org/10.24918/cs.2016.15 (26 April 2018, date last accessed).

[bib25] Pelletreau KN, Knight JK, Lemons P A faculty professional development model that improves student learning, encourages student-centered instructional practices, and works for geographically separated faculty. CBE-Life Sci Educ(submitted,reviewed with minor revisions). 2018;17:es5. 29749849 10.1187/cbe.17-12-0260 PMC5998327

[bib26] Pfund C, Miller S, Brenner K Summer institute to improve university science teaching. Science 2009;324:470–1. 19390031 10.1126/science.1170015

[bib27] Prevost LB, Smith MK, Knight JK Using student writing and lexical analysis to reveal student thinking about the role of stop codons in the central dogma. Cell Biol Educ 2016;15:ar65. 10.1187/cbe.15-12-0267 PMC5132362 27909016

[bib28] President's Council of Advisors on Science and Technology Engage to Excel: Producing One Million Additional College Graduates with Degrees in Science, Technology, Engineering, and Mathematics. Report to the President (Executive Office of the President, Washington DC, 2012) (26 April 2018, date last accessed).

[bib29] Sestero C, Tinsley H, Ye ZH Using the cell engineer/detective approach to explore cell structure and function. CourseSource 2014;1–5. doi: 10.24918/cs.2014.7.

[bib30] Silverthorn DU, Thorn PM, Svinicki MD It's difficult to change the way we teach: lessons from the Integrative Themes in Physiology curriculum module project. Adv Physiol Educ 2006;30:204–14. 17108248 10.1152/advan.00064.2006

[bib31] Smith MK, Wood WB, Knight JK The genetics concept assessment: a new concept inventory for gauging student understanding of genetics. Cell Biol Educ 2008;7:422–30. 10.1187/cbe.08-08-0045 PMC2592048 19047428

[bib32] Smith MK, Wenderoth MP, Tyler M The teaching demonstration: what faculty expect and how to prepare for this aspect of the job interview. Cell Biol Educ 2013;12:12–8. 10.1187/cbe.12-09-0161 PMC3587851 23463224

[bib33] Smith MK, Jones FHM, Gilbert SL The classroom observation protocol for undergraduate stem (COPUS): a new instrument to characterize university STEM classroom practices. Cell Biol Educ 2013;12:618–27. 10.1187/cbe.13-08-0154 PMC3846513 24297289

[bib34] Smith MK, Toth ES, Borges K Using place-based economically relevant organisms to improve student understanding of the roles of carbon dioxide, sunlight, and nutrients in photosynthetic organisms. CourseSource 2018 https://doi.org/10.24918/cs.2018.1 (26 April 2018, date last accessed).

[bib35] Stains M, Harshman J, Barker MK Anatomy of STEM teaching in American universities: a snapshot from a large-scale observation study. Science 2018;359:1468–70. 29599232 10.1126/science.aap8892 PMC6310123

[bib36] Sursock A Trends 2015: Learning and Teaching in European Universities Brüssel European University Association. 2015. http://www.eua.be/Libraries/publications-homepage-list/EUA_Trends_2015_web (30 January 2018, date last accessed).

[bib37] Wood WB, Handelsman J Meeting report: The 2004 national academies summer institute on undergraduate education in biology. Cell Biol Educ 2004;3:215–7. 15592593 10.1187/cbe.04-07-0057 PMC533119

